# Critical Appraisal of the Quality of Clinical Practice Guidelines for Stress Ulcer Prophylaxis

**DOI:** 10.1371/journal.pone.0155020

**Published:** 2016-05-06

**Authors:** Zhi-Kang Ye, Ying Liu, Xiang-Li Cui, Li-Hong Liu

**Affiliations:** Department of Pharmacy, Beijing Chao-Yang Hospital, Capital Medical University, Beijing, China; University Hospital Llandough, UNITED KINGDOM

## Abstract

**Background and Objective:**

Inappropriate use of stress ulcer prophylaxis (SUP) is common in many hospitals. High-quality clinical practice guidelines (CPGs) produce better patient outcomes and promote cost-effective clinical care. Thus, the objective of this study was to evaluate the quality of CPGs for SUP.

**Methods:**

A search was conducted for SUP CPGs using PubMed, Embase, China National Knowledge Infrastructure (CNKI), Chinese Biomedical Literature database (CBM), guideline websites and Google (until March 1, 2015). The quality of CPGs was independently assessed by two assessors using the Appraisal of Guidelines for Research & Evaluation II (AGREE II) instrument, and the specific recommendations in the CPGs were summarized and evaluated.

**Results:**

A total of 7 CPGs for SUP were included. The highest median scores were in the clarity of presentation domain (89%), and the lowest median scores were in the editorial independence domain (0%). The rigor of development, stakeholder involvement, and applicability domains all scored below 40%. The specific recommendations for SUP varied, and the recommendations were inconsistent with the supporting evidence.

**Conclusions:**

The overall quality of CPGs for SUP was relatively low, and no specific SUP CPG can be recommended. Not only should the AGREE II instrument be used to determine the quality of CPGs, but also the recommendations should be appraised based on supporting evidence, which would contribute to the development of high-quality CPGs.

## Introduction

Stress ulcer prophylaxis (SUP) is commonly used to decrease gastrointestinal bleeding in both critically and non-critically ill patients [1–3]. To help clinicians appropriately use SUP, several organizations have developed clinical practice guidelines (CPGs) for SUP.

Many studies suggested that the inappropriate use of SUP, based on these guidelines, is common in hospital [4–12]. CPGs are “statements that include recommendations intended to optimize patient care. They are informed by a systematic review of evidence and an assessment of the benefits and harms of alternative care option” [13]. High-quality CPGs provide effective recommendations, produce better patient outcomes and promote cost-effective clinical care [14]. Both high AGREE II domain scores and high-quality recommendations are needed to produce excellent CPGs.

To the best of our knowledge, there has been no evaluation of SUP guidelines. Thus, we conducted this study to evaluate the quality of a group of international CPGs for SUP and to help develop, update or improve SUP guidelines, and to help clinicians reduce the inappropriate use of SUP.

## Methods

### Literature Search

We searched PubMed, Embase, China National Knowledge Infrastructure (CNKI) and Chinese Biomedical Literature Database (CBM) for SUP guidelines (until March 1, 2015). The text words and Medical Subject Headings (MeSH) terms were as follows: (guideline or guidelines or consensus) and “stress ulcer prophylaxis”. The CPGs search was conducted on major guideline websites, including the National Guideline Clearinghouse, National Institute for Health and Clinical Excellence, Scottish Intercollegiate Guidelines Network, Guidelines International Network and China Guideline Clearinghouse. The search term was “stress ulcer prophylaxis”. Google was also searched using the terms “guideline” and “stress ulcer prophylaxis”, and we reviewed the first 100 results. No restriction on language was applied.

### Selection of Guidelines

CPGs for SUP included those that provided clinical recommendations and strategies to assist health care practitioners in making decisions and those that were endorsed by medical specialty associations or relevant professional societies. We excluded guidelines that were not original, were duplications, or were explanations of CPGs.

### Quality Evaluation

Two assessors, one with experience in developing and evaluating guidelines (Z.K.Y) and another assessor (Y.L), used the online training tools recommended by the AGREE collaboration before conducting appraisals. They independently evaluated the included guidelines using the AGREE II instrument [15], which consists of 23 items organized into six domains. Each item was rated on a seven-point scale from 1 (strongly disagree) to 7 (strongly agree). To resolve discrepancies between the two assessors, we referred to the method used in a previous study: if the scores assigned by the assessors differed by one point, the lower score was used; if they differed by two points, they were averaged; and if they varied by three points or more, a consensus was reached after a discussion [16]. A scaled domain score was calculated as follow: (obtained score—minimum possible score)/(maximum possible score—minimum possible score). The overall recommendation about the included CPGs was based on the scores for each item and the quality of the CPGs’ recommendations.

After determining the quality of SUP guidelines using the AGREE II instrument, the specific recommendations made in the included guidelines were summarized and evaluated, including indications for SUP, agents for SUP and duration of prophylaxis.

## Results

### Study Selection

**Fig 1:**
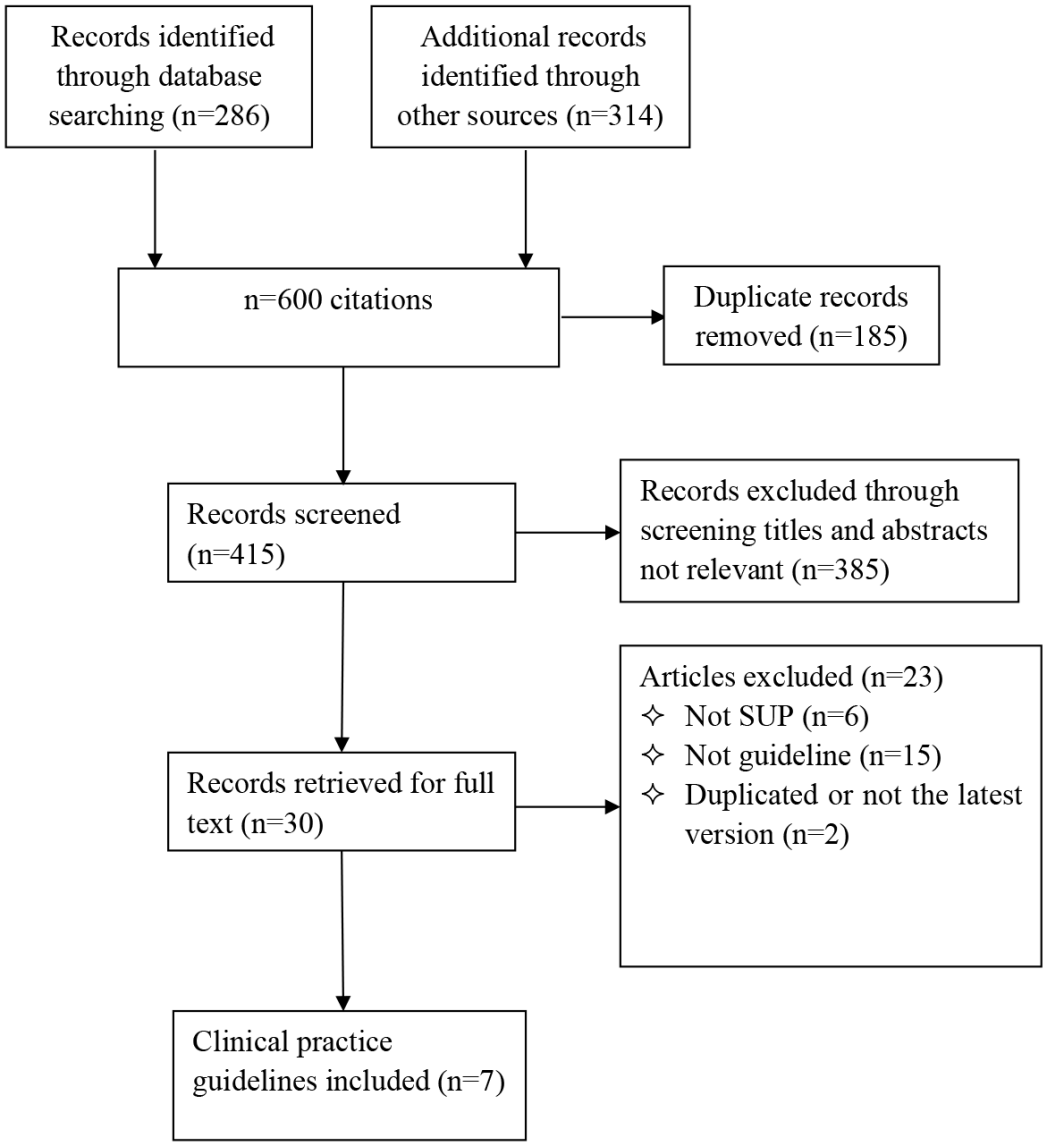
Flowchart for systematically searching and selecting the guidelines.

A total of 600 records were identified, of which, 185 were duplications, 385 were either not relevant to SUP or not guidelines after screening titles and abstracts, and the remaining 30 records were retrieved for full text. Finally, 7 CPGs (ASHP [17], EAST [18], ORMC [19], VUMC [20], DASAIM [21], NMJC [22], KAUH [23]) were included (see Fig 1). The demographic characteristics for included guidelines are presented in Table 1. All CPGs were published between 1999 and 2013. The ASHP and DASAIM CPGs were found in medical databases [17,21], the EAST CPG was found on a guideline website [18], and the remaining CPGs were found through Google searches. The DASAIM CPG rated the quality of evidence and graded the strength of recommendations using the Grading of Recommendations Assessment, Development and Evaluation (GRADE) approach, and the DASAIM CPG was developed specifically for intensive care unit patients [21].

**Table 1:** Characteristics of clinical practice guidelines. ASHP: American Society of Health-System Pharmacists; EAST: Eastern Association for the Surgery of Trauma; ORMC: Orlando Regional Medical Center; VUMC: Vanderbilt University Medical Center; DASAIM: Danish Society of Anesthesiology and Intensive Care Medicine; DSIT: Danish Society of Intensive Care Medicine; EB: Editorial Board; NMJC: National Medical Journal of China; DIC: Drug Information Center; KAUH: King Abdullah University Hospital.

Title	Year of publication	Country/Region	Level of development	Organization	Number of authors	Number of references
ASHP Therapeutic Guidelines on Stress Ulcer Prophylaxis (ASHP) [17]	1999	America	National	ASHP	10	278
Practice Management Guidelines for Stress Ulcer Prophylaxis (EAST) [18]	2008	America	Regional	EAST	8	58
Stress Ulcer Prophylaxis (ORMC) [19]	2011	America, Orlando	Regional	ORMC	NR	14
Gastrointestinal Stress Ulcer Prophylaxis Guideline (VUMC) [20]	2005	America, Tennessee	Regional	VUMC	2	13
Guideline for Stress Ulcer Prophylaxis in the Intensive Care Unit (DASAIM) [21]	2014	Denmark	National	DASAIM/DSIT	7	28
Consensus Review for Stress Ulcer Prophylaxis and Treatment (NMJC) [22]	2002	China	National	EB of NMJC	10	NR
Stress Ulcer Prophylaxis (SUP) Guideline (KAUH) [23]	NR	Jordan	Regional	DIC of KAUH	1	5

### Scope and Purpose

The domain-standardized scores for SUP CPGs and overall recommendation were presented in Table 2. The median score for the scope and purpose domain was 67% (range 39–100%). Only the ORMC and NMJC CPGs scored below 60% in this domain [19,22]. Most guidelines clearly described their overall objectives, health questions and target populations.

**Table 2:** AGREE II domain-standardized scores for CPGs on SUP.

Guideline	Scope and Purpose (%)	Stakeholder Involvement (%)	Rigor of Development (%)	Clarity of Presentation (%)	Applicability (%)	Editorial Independence (%)	Overall Assessment
ASHP [17]	83	61	65	94	54	17	Recommended with modifications
EAST [18]	67	39	44	89	29	0	Recommended with modifications
ORMC [19]	56	17	27	83	38	0	Not recommended
VUMC [20]	72	33	12	89	38	0	Not recommended
DASAIM [21]	100	33	77	94	38	33	Recommended with modifications
NMJC [22]	39	39	6	67	21	0	Not recommended
KAUH [23]	61	39	19	83	46	0	Not recommended
Median (Range)	67 (39–100)	39 (17–61)	27 (6–77)	89 (67–94)	38 (21–54)	0 (0–33)	

### Stakeholder Involvement

The median score for the stakeholder involvement domain was 39% (range 17–61%). Only the AHSP CPG scored above 60% for this domain [17]. No guideline considered the views and preferences of the target population, and no guideline clearly described their members’ roles in the guideline development process. No methodology experts or health economists were included in the guideline development group.

### Rigor of Development

The median score for the rigor of development domain was 27% (range 6–77%). The ASHP and DASAIM CPGs scored above 60% [17,21]. Only the AHSP and DASAIM CPGs clearly described systematic methods of searching for evidence [17,21]. Only the DASAIM CPG clearly described methods for formulating recommendations and for conducting external reviews [21]. Only the DASAIM CPG used the GRADE approach to developing guidelines and reporting the recommendations based on an underlying systematic review [21]. No guideline described their procedures for updating guidelines.

### Clarity of Presentation

The median score for the clarity of presentation domain was 89% (range 67–94%). All guidelines scored above 60%. Most guidelines provided specific, unambiguous and easily identifiable recommendations.

### Applicability

The median score for the applicability domain was 38% (21–54%). No guideline scored above 60%. No guideline systematically described the facilitators and barriers of its applications very well. Most guidelines did not sufficiently consider the costs of applying their recommendations, and no guideline involved a health economist in finding and analyzing cost information.

### Editorial Independence

The median score for the editorial independence domain was 0% (0–33%). No guideline clearly provided funding information. Only the DASAIM CPG described the competing interests of the guideline development group members [21]. The AHSP CPG described the method by which potential competing interests were sought, but competing interests results were not provided [17]. Other guidelines did not provide information about competing interests.

### Specific Recommendations

#### Indications for SUP

In Table 3, indications for SUP was described. Six CPGs (ASHP, EAST, ORMC, VUMC, NMJC and KAUH) recommended mechanical ventilation, coagulopathy and traumatic brain injury as the indications for SUP. Five CPGs (ASHP, ORMC, VUMC, NMJC and KAUH) recommended that a history of gastrointestinal ulceration or bleeding within one year was an indication for SUP. Five CPGs (ASHP, EAST, ORMC, NMJC and KAUH) recommended that major burn injury was an indication for SUP. Four CPGs (EAST, ORMC, VUMC and NMJC) recommended that sepsis was an indication for SUP, while two CPGs (ASHP and KAUH) recommended that sepsis was a minor risk factor for SUP. Three CPGs (ASHP, EAST and NMJC) recommended that multi-trauma was an indication for SUP. Four CPGs (ASHP, EAST, VUMC and KAUH) recommended that receiving high-dose corticosteroids was a minor risk for SUP. The DASAIM CPG did not make any recommendation about indications for SUP because of insufficient evidence. When we evaluated the original studies supporting these recommendations, we found no high-quality study on indications for SUP. Thus, recommendations on indications for SUP were not very consistent across guidelines.

**Table 3:** Specific recommendations from CPGs. NR: not reported; GI: gastrointestinal.

Specific recommendations	ASHP	EAST	ORMC	VUMC	DASAIM	NMJC	KAUH
Indications for SUP							
Mechanical ventilation	√	√	√	√	NR	√	√
Coagulopathy	√	√	√	√	NR	√	√
A history of GI ulceration or bleeding within one year	√	NR	√	√	NR	√	√
Traumatic brain injury	√	√	√	√	NR	√	√
Major burn injury	√	√	√	NR	NR	√	√
Sepsis	Minor risk	√	√	√	NR	√	Minor risk
Multi-trauma	√	√	NR	NR	NR	√	NR
High-dose corticosteroids	Minor risk	Minor risk	√	Minor risk	NR	NR	Minor risk
Agents for SUP	Antacids, H_2_RAs, sucralfate	H_2_RAs, PPIs, cytoprotective agents	H_2_RAs, PPIs	Famotidine, PPIs	PPIs	PPIs, H_2_RAs, antacids, mucosal protective agents	H_2_RAs, PPIs, sucralfate, antacids
Duration of prophylaxis	Until no risk factors	Until not receiving mechanical ventilation or not in ICU, or able to tolerate enteral nutrition	Until no risk factors, or able to tolerate enteral feeding	Until no high risk factors, or able to tolerate enteral feeding	NR	NR	Until no high risk factors

#### Agents for SUP

Recommendations on agents for SUP were not consistent across these guidelines. The DASAIM CPG recommended using proton pump inhibitors (PPIs) rather than histamine 2 receptor antagonists (H_2_RAs) for SUP, five CPGs (EAST, ORMC, VUMC, NMJC and KAUH) recommended using both PPIs and H_2_RAs, and the ASHP CPG did not recommended using PPIs. Three CPGs (ASHP, NMJC and KAUH) recommended using antacids for SUP, while two CPGs (ASHP, KAUH) recommended using sucralfate for SUP. The EAST CPG recommended cytoprotective agents for SUP, and the NMJC CPG recommended mucosal protective agents.

The DASAIM CPG recommended using PPIs rather than H_2_RAs for SUP based on one published systematic review, which suggesting that PPIs were more effective than H_2_RAs at reducing clinically important upper gastrointestinal bleeding (RR = 0.36, 95%CI 0.19–0.68 P = 0.002). However, after excluding trials at high risk or unclear risk of bias, the results suggested that there was no significant difference between PPIs and H_2_RAs (RR = 0.60, 95%CI 0.27–1.35 P = 0.21) [24]. Therefore, the recommendations of included CPGs were not consistent with the supporting evidence.

#### Duration of Prophylaxis

Recommendations on duration of prophylaxis are moderately consistent. Five CPGs (ASHP, EAST, ORMC, VUMC and KAUH) recommended that prophylaxis should be discontinued when there was no risk factor for SUP, three CPGs (EAST, ORMC, VUMC) recommended that prophylaxis be discontinued when the patient can tolerate enteral feeding, and two CPGs (DASAIM and NMJC) did not provide recommendations on duration of prophylaxis. When we evaluated the original studies supporting the recommendations, we found no-high quality studies on duration of prophylaxis.

### Overall Assessment

The DASAIM, ASHP and EAST CPGs were recommended with modifications [17,18,21], while the remaining CPGs were not recommended [19,20,22,23].

## Discussion

This is the first study to systematically evaluate the quality of SUP guidelines. The overall quality of these CPGs was relatively low; the clarity of presentation domain showed the highest scores, while the editorial independence domain showed the lowest scores. Not only should the AGREE II instrument be used to determine the quality of CPGs, but also the recommendations should be appraised based on supporting evidence.

These guidelines had high clarity of presentation scores, indicating that this domain was more easily achieved than other domains. The editorial independence domain was poorly described. No guideline provided funding information, and only two guidelines described competing interests. Perhaps guideline developers do not like disclosing their funding information, or perhaps they do not realize the importance of conflict of interest disclosures and management. Studies have shown that financial conflicts of interest are prevalent among CPGs in a variety of clinical areas [25,26], and some evidence suggests that such financial conflicts of interest may affect guideline recommendations [27]. Therefore, guideline developers should strongly emphasize the editorial independence domain.

The rigor of development, stakeholder involvement and applicability domains all scored below 40%, and there were serious methodological flaws in these three domains. The median score for the rigor of development domain was 27%. Most guidelines described neither systematic methods for evidence searching nor methods for formulating recommendations very well. Only the DASAIM CPG indicated that the guideline was reviewed by external experts, and no guideline provided procedures for updating guidelines. The median score for the applicability domain was 38%. Most guidelines did not consider the potential resource implications of applying recommendations, nor did they pay proper attention to potential barriers to guideline implementation.

The median score for the stakeholder involvement domain was 39%. No guideline considered the views and preferences of target populations; however, the involvement of patients in decision making might promote patient guideline adherence and improve clinical outcomes [28]. Most guidelines did not provided clear information on guideline development group members, especially on the members’ roles in the guideline development group. No guideline involved methodological experts or health economists, which explains why all of these guidelines scored low in the rigor of development domain. The guideline manuals published by World Health Organization and the National Institute for Health and Clinical Excellence both recommended that methodologists be involved in guideline development [29,30]. Guideline developers should pay more attention to the composition of guideline development groups and incorporate patient preferences. The median score for the scope and purpose domain was 67%, and most guidelines described their overall objectives, specific health questions and target populations well.

The specific recommendations made in the included CPGs varied, probably due to the lack of high-quality studies on the indications for SUP and the duration of prophylaxis. Guideline developers failed to critically appraise the validity of the evidence, which led to the inconsistency between the recommendations and the supporting evidence. The DASAIM CPG rated the quality of evidence and graded the strength of recommendations using the GRADE approach, and recommendations were formulated based on a systematic review, thus scoring the highest in the rigor of development. However, high AGREE II domain scores do not imply that a guideline should be recommended. When we appraised the DASAIM CPG, we found that some recommendations were not consistent with the supporting evidence. The outcomes of trials with low risk of bias did not suggest that PPIs were better than H_2_RAs. Guideline developers should critically appraise the validity of systematic reviews and other sources of evidence formulating recommendations.

All potentially relevant studies were retrieved by searching medical databases, five guideline websites and Google. This study also has limitation. The AGREE II instrument established an evaluation system for guideline development and reporting, but the appraisal of guideline recommendations is not stated.

In conclusion, the overall quality of CPGs for SUP was relatively low, no specific SUP CPG can be recommended to guide clinical practice. Not only should the AGREE II instrument be used to determine the quality of CPGs, but also the recommendations should be appraised based on supporting evidence, which would contribute to the development of high-quality SUP CPGs.

## Supporting Information

S1 Fig PRISMA Flow Diagram. (DOC)

S1 Table PRISMA Checklist. (DOC)
