# Supplementary material for: Critical Appraisal of the Quality of Clinical Practice Guidelines for Stress Ulcer Prophylaxis
Source: PLoS One. 2016 May 6;11(5):e0155020. doi: 10.1371/journal.pone.0155020 (PMC4859569; doi:10.1371/journal.pone.0155020)
Supplement: S1 Fig — (DOC) [file pone.0155020.s001.doc]

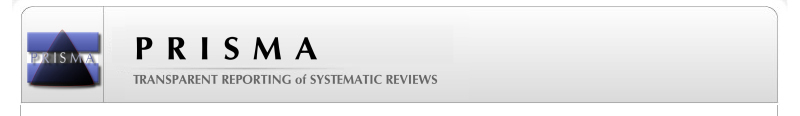
**PRISMA 2009 Flow Diagram**

**Screening**

**Included**

**Eligibility**

**Identification**

Records identified through database searching
(n =286 )

Additional records identified through other sources
(n =314 )

Records after duplicates removed
(n =415 )

Records screened
(n =415 )

Records excluded
(n =385 )

Full-text articles assessed for eligibility
(n =30 )

Full-text articles excluded, with reasons
(n = 23 )

Studies included in qualitative synthesis
(n = 7 )

Studies included in quantitative synthesis (meta-analysis)
(n = )
